# Physics-Guided Learning for Monocular Visual Object Localization in Indoor Environments

**DOI:** 10.3390/s26154674

**Published:** 2026-07-23

**Authors:** Haorui Ge, Luzheng Bi, Weijie Fei, Jiarong Wang

**Affiliations:** The School of Mechanical Engineering, Beijing Institute of Technology, Beijing 100081, China; 3120245299@bit.edu.cn (H.G.); bhxblz@bit.edu.cn (L.B.); bitfwj@bit.edu.cn (W.F.)

**Keywords:** indoor short-range object localization, monocular vision, physics-guided learning, visual perception

## Abstract

Accurate object localization is essential for enabling autonomous operation of indoor robotic systems. As a low-cost, compact, and flexibly deployable solution, monocular visual object localization (MVOL) is highly applicable to lightweight embedded robotic platforms. However, conventional data-driven MVOL methods suffer from inherent limitations of 2D-to-3D ill-posed mapping, which is caused by the incapability of constraining the spatial physical logic of real scenes, resulting in severe depth ambiguity, inaccurate scale estimation, and physically unreasonable predictions. To address these issues, this paper proposes a novel physics-guided monocular visual localization framework termed *PC-IMVL* for indoor scenarios. The PC-IMVL integrates deep visual perception with embedded physical modeling, which explicitly introduces spatial physical constraints into the network optimization process and builds a physical consistency-aware loss function to regularize 3D position and pose estimation. Combined with a lightweight tailored architecture, the framework enables efficient and reliable embedded deployment. Offline experiments and real-world online tests validate the effectiveness of the proposed method. PC-IMVL yields average absolute errors (AE) of 0.095–0.333 m, reducing the localization error of early fusion methods by more than 50%. Within a working distance of 3–4 m, it achieves a relative error (RE) of 2.4% and a horizontal viewing angle error (VAE) below 2°, outperforming existing state-of-the-art MVOL methods. The effectiveness of the physical guidance mechanism is verified. This work provides a practical high-precision localization solution for embedded indoor robotic systems.

## 1. Introduction

Indoor robotic systems are being increasingly deployed in home service, industrial maintenance, and high-risk operation scenarios, where reliable three-dimensional (3D) object localization serves as a fundamental prerequisite for autonomous navigation and manipulation tasks [1]. While deep learning has significantly advanced two-dimensional (2D) object detection and feature representation techniques [2], precise and physically consistent 3D localization remains a critical bottleneck for lightweight embedded robots operating in unstructured, cluttered, and low-light indoor environments [3]. Compared with expensive LiDAR devices, bulky multi-camera systems, and complex multi-sensor fusion modules, monocular visual object localization (MVOL) provides a low-cost, compact, and highly deployable perception solution, making it the most suitable paradigm for resource-constrained indoor robotic platforms.

Compared with MVOL, LiDAR can directly output metric depth without scale ambiguity, yet it suffers from high hardware cost, large volume, and poor robustness under heavy occlusion, which limits its deployment on miniature lightweight robots. Multi-camera stereo systems leverage calibrated baselines to recover depth via triangulation; however, they require strict hardware synchronization, extra installation space, and precise offline calibration. Their localization performance also degrades drastically when targets are far beyond the effective baseline range. In contrast, monocular cameras eliminate the above hardware overhead, but they lack parallax constraints, resulting in an inherent ill-posed 2D-to-3D mapping problem unique to single-view vision.

Despite its prominent deployment advantages, conventional data-driven MVOL methods suffer from inherent technical defects rooted in the ill-posed 2D-to-3D mapping process. Existing monocular localization solutions generally rely on category-level size priors, pre-established 3D object models, or multi-view triangulation to recover metric depth and scale information, including traditional PnP and SLAM-based geometric methods [4,5] and modern 3D bounding box detection approaches such as FCOS3D, MonoDLE, and SMOKE [6,7,8]. Data-driven networks merely learn statistical appearance correlations from training datasets without explicit physical and geometric constraints, inevitably producing severe depth ambiguity, scale distortion, and physically unreasonable predictions. Such abnormal outputs, including object floating and spatial penetration, greatly degrade localization accuracy and robustness in close-range, occluded, and low-illumination indoor scenarios [9]. Although recent monocular depth estimation methods can generate dense relative depth cues, they fail to produce metrically accurate and physically consistent 3D coordinates without instance-level prior constraints [10], which severely limits the practical application of monocular localization systems.

In contrast to prior-dependent and multi-view-based localization paradigms, this work targets a more practical and challenging object-prior-free single-view MVOL setting. For clarity, the term object-prior-free herein exclusively denotes that the pipeline does not require object dimension templates, CAD models, scene geometric scale priors, category size statistics, or pre-built target 3D models during inference. Camera intrinsic calibration parameters, generic pre-trained detection and depth backbones, and training annotations are not regarded as object-specific priors in this definition. In this scenario, robots are required to infer metric 3D coordinates of arbitrary target objects solely from a single RGB image, without relying on object dimension templates, CAD models, scene geometric priors, sequential frame matching, or active viewpoint adjustment for triangulation. Such a strict single-shot and object-prior-free constraint conforms to real indoor robot operating conditions, where novel unknown objects lack category-level supervision, miniature embedded devices are only equipped with a single camera, and narrow working spaces and real-time task requirements prevent baseline construction and temporal information fusion. In this case, traditional localization schemes are essentially infeasible. LiDAR-based methods suffer from high cost and limited robustness against occlusion [11]. Multi-view stereo and SfM methods require calibrated baselines or sequential camera motion for triangulation [12]. Active vision relies on controllable camera movement to construct spatial baselines [13]. Multi-sensor fusion introduces excessive hardware and calibration overhead [14]. Moreover, early fusion-based monocular methods, such as Pseudo-LiDAR and DD3D, suffer from severe error accumulation and scale drift when lacking object prior constraints [15,16].

To address the above technical gaps, this paper proposes a Physics-Constrained Indoor Monocular Visual Localization (PC-IMVL) method for object-prior-free single-view 3D object localization. Different from conventional purely data-driven monocular methods that rely on category priors or multi-frame triangulation, PC-IMVL explicitly embeds camera geometric rules and spatial physical consistency constraints into the entire network learning pipeline, constructing a physical regularization loss mechanism to eliminate scale ambiguity and physical invalid predictions. Equipped with a lightweight architecture tailored for embedded platforms, the proposed method realizes accurate metric 3D coordinate estimation through single-shot inference without any prior knowledge or triangulation operations. Extensive experiments on embedded devices and real-world online tests demonstrate that PC-IMVL achieves superior localization performance in complex indoor short-range scenarios, with significantly improved accuracy, physical rationality, and deployment practicability compared with state-of-the-art MVOL methods. This work provides an effective and reliable perception solution for high-precision visual localization of embedded indoor robots. The contributions of this work can be summarized as follows:

- This work constructs a novel physics-guided monocular localization framework that integrates geometric physical constraints into network optimization, effectively solving the physical inconsistency and scale ambiguity problems of traditional data-driven MVOL methods.
- The proposed object-prior-free single-view localization paradigm eliminates the dependence on object size templates, scene geometric priors, and multi-view triangulation, which greatly enhances the adaptability for complex and unknown indoor scenarios.
- The lightweight embedded-friendly architecture ensures high-precision metric 3D coordinate prediction while maintaining low inference overhead, realizing a favorable balance among localization accuracy, physical rationality, and deployment efficiency for practical indoor robotic applications.

## 2. Related Works

### 2.1. Existing Methods

We categorize monocular 3D localization methods by their sensing configuration, prior requirements, and output modalities, as shown in Table 1. We further review recent physics-informed and geometry-constrained monocular learning approaches and find that these existing physical regularization schemes are mostly decoupled from instance-level 3D coordinate regression, dependent on multi-view or temporal input, or still demand object size priors to recover metric scale. Different from both traditional pipelines and recent physics-guided vision work, our paper focuses on single-shot, single-view metric coordinate estimation without any object or scene prior, a paradigm with fully end-to-end embedded pinhole geometric constraints that is fundamentally distinct from all compared approaches.

#### 2.1.1. Multi-View Triangulation Methods

Structure-from-Motion (SfM) and visual SLAM reconstruct scene 3D geometry via multi-view triangulation, which hinges on temporally consecutive camera movements with known trajectories or pre-calibrated stereo baselines, as validated in prior literature [5,12,18]. Mathematically, these paradigms resolve ambiguous depth measurements relying on the triangulation principle formulated as:

(1) $$\mathrm{Depth}=\frac{\mathrm{Baseline}\times \mathrm{Focal}\;\mathrm{Length}}{\mathrm{Disparity}}$$

Despite their prevalent usage, such triangulation-based pipelines come with inherent drawbacks incompatible with our task setup. Stereo-based implementations demand meticulously calibrated baseline parameters and rigorous frame synchronization between paired cameras, while monocular SLAM cannot operate robustly without adequate translational camera parallax and would collapse facing static scenes or pure rotational camera motion. Moreover, the multi-frame data aggregation inherent to classic SfM and SLAM inevitably induces computational latency that prohibits low-latency real-time deployment; another critical bottleneck lies in the inherent scale ambiguity of monocular SfM, where reconstructed scene geometry is defined only up to an unknown scale and extra auxiliary priors or external sensors are mandatory to attain metric localization. Collectively, the above prerequisites and drawbacks contradict our design target of single-shot passive localization. Different from triangulation-dependent pipelines, our proposed PC-IMVL abandons multi-view triangulation completely and directly regresses metric depth solely from single RGB images with the supervision of embedded physical constraints.

#### 2.1.2. PnP and Model-Based Methods

Perspective-n-Point (PnP) solvers and feature-based methods establish 2D-3D correspondences with known object models to recover 6DoF pose [4,17,21]. While accurate with perfect models (<0.05 m) [21], they require per-instance CAD models or dense point clouds, violating our object-prior-free constraint. These methods are inapplicable to novel objects in unstructured environments.

#### 2.1.3. 3D Bounding Box Detectors

Representative monocular 3D object detection approaches, including FCOS3D [6], MonoDLE [7], SMOKE [8], and GUP Net [22], regress complete 3D bounding box descriptions consisting of object center location, physical dimensions (w,h,l), and heading orientation from individual RGB inputs. Nevertheless, this bounding-box-centered formulation introduces several intrinsic drawbacks that hinder its deployment toward our target setting. First, the estimated object dimensions suffer from inherent scale ambiguity, such that recovered geometry is defined only in a relative scale and extra object-size priors or post-processing operations are indispensable to achieve metric localization [7]. In addition, these frameworks cannot directly output absolute metric coordinates (X,Y,Z); extra empirical bottom-center projection rules are required to convert predicted box centers into practically usable spatial coordinates for downstream robotic manipulation [6]. Furthermore, existing detectors are predominantly tailored for autonomous-driving scenarios with closed-set predefined object classes (e.g., vehicles and pedestrians), whose statistically averaged physical sizes can be exploited for scale calibration [7]. Owing to the above restrictions, conventional 3D bounding box detection pipelines fail to satisfy the requirements of our indoor localization task involving unseen arbitrary objects with unknown sizes.

#### 2.1.4. Early Fusion Methods (EFM)

Existing early fusion methods (EFM) follow the prevalent depth-then-geometry pipeline widely adopted in resource-limited robotic localization tasks [15,16,19]. Different from multi-view triangulation-based schemes, these approaches achieve single-view metric coordinate recovery by cascading monocular depth prediction and pinhole camera geometric projection, whose inference pipeline is illustrated as follows:

(2) $$\mathrm{RGB}\;(\mathrm{single}\;\mathrm{view})\overset{\mathrm{Monocular}\;\mathrm{Depth}}{\to }Z\overset{\mathrm{Pinhole}\;\mathrm{Model}}{\to }\left(X,Y,Z\right)$$

Typical EFM implementations include Pseudo-LiDAR [15], which transforms predicted depth maps into pseudo-LiDAR point clouds for 3D detection, DD3D [16], which adopts a depth-pre-training and detection-finetuning paradigm, and PatchNet [19], which enhances depth feature representation to boost single-view 3D localization performance. Despite their effectiveness in conventional single-view 3D perception tasks, such depth-guided fusion pipelines suffer from three inherent limitations that fail to meet our object-prior-free indoor localization requirements. First, monocular depth estimation inherently suffers from scale ambiguity, such that accurate metric coordinate recovery relies heavily on category-averaged object dimensions or data-driven learned scale priors [15,16], which violates the object-prior-free setting of our task. Second, depth prediction inevitably introduces non-negligible estimation errors (5–15% for typical indoor scenarios [23]), which are further amplified in a quadratic manner during geometric projection, especially for objects captured under oblique viewing angles [19]. Third, the decoupled optimization of depth estimation and geometric projection eliminates end-to-end error calibration, making the pipeline unable to compensate for cumulative prediction and projection errors adaptively.

To establish a comprehensive and strong baseline for fair comparison, we implement a state-of-the-art EFM pipeline tailored for our indoor task. Our EFM baseline integrates DepthAnything-V2 [10] for high-precision monocular depth prediction, YOLOv11 [24] for object detection, and standard pinhole projection with predefined category-averaged dimension priors (1.7 m for person, 0.5 m for box, and 0.3 m for unmanned ground vehicle (UGV)). This optimized implementation constitutes a powerful prior-aware EFM baseline that completely relies on single-view depth estimation instead of multi-view triangulation, serving as a rigorous contrast to our object-prior-free end-to-end framework.

#### 2.1.5. Pure Deep Learning Regression

Different from geometry-guided and depth-fusion paradigms, pure deep learning (DL) regression methods abandon explicit geometric modeling entirely and directly infer metric object coordinates from individual RGB images without requiring multi-view triangulation. The overall inference pipeline of such end-to-end regression schemes is formulated as:

(3) $$\mathrm{RGB}\;(\mathrm{single}\;\mathrm{view})\overset{\mathrm{CNN}}{\to }f\overset{\mathrm{MLP}}{\to }{\left(X,Y,Z\right)}_{\mathrm{direct}}$$

Existing literature has extensively explored such direct coordinate regression solutions for single-view 3D localization. Tatarchenko et al. [20] systematically analyzed the implicit learning mechanism of single-view reconstruction networks, while subsequent studies further validated the feasibility of direct coordinate regression for indoor object localization tasks [25]. Nevertheless, pure DL regression pipelines exhibit inherent defects stemming from the absence of explicit geometric supervision, restricting their practical deployment in precise robotic localization. Primarily, these networks merely fit implicit data mappings from training samples without embedding physical camera geometric constraints, which frequently yields unreasonable predictions including floating objects and disordered depth relationships that contradict real-world physical rules [20]. Moreover, the lack of geometric regularization leads to severe coordinate drift and degraded physical consistency in predicted results, particularly when encountering unseen domain shifts in complex indoor scenarios [25]. Beyond inaccurate predictions, the fully black-box regression paradigm also suffers from poor interpretability, which substantially hinders model debugging, performance analysis, and practical safety certification for robotic applications.

To quantitatively analyze the contribution of embedded physical constraints in our framework, we construct a pure DL regression baseline based on the proposed PC-IMVL architecture. Specifically, we remove all intermediate physical constraint branches and the corresponding physical loss $L_{\mathrm{phy}}$, retaining only the spatial regression loss $L_{\mathrm{spatial}}$ to implement standard end-to-end coordinate regression. For fair and rigorous ablation comparison, the core network configurations, including the ResNet-34 backbone and MLP regression heads, are completely consistent with the original PC-IMVL model, which effectively isolates the performance improvement brought by physical constraint modeling.

#### 2.1.6. Recent Physics-Informed and Geometry-Constrained Monocular Vision Methods

A growing body of recent work integrates physical and geometric regularizers into monocular visual pipelines, yet these existing constraint designs cannot be directly migrated to our object-prior-free single-shot indoor localization task. Many geometry-constrained depth estimation methods only apply surface normal or smoothness loss on dense pixel-level depth maps without targeting instance-level target 3D coordinate regression, and their geometric penalties operate independently of final spatial position output, forming a decoupled optimization pipeline that inevitably accumulates projection error. Some motion-physics monocular localization methods introduce kinematic rules for dynamic objects, but they require sequential video frames during training and rely on temporal multi-frame constraints which conflict with our single-shot inference setting. Recent large-scale geometry-aware transformer frameworks build cross-view consistent loss for outdoor mapping, whose physical constraints are constructed based on multi-view triangulation and long video sequences, lacking lightweight differentiable pinhole intermediate variables optimized for close-range indoor robots. Their loss functions only combine depth consistency and photometric terms, without the segmented angular penalty and cross-coupling mechanism we design to balance spatial positioning accuracy and viewing angle rationality. Unlike these existing schemes that treat physical rules as auxiliary post-hoc regularization, our PC-IMVL embeds interpretable geometric variables including distance, horizontal and vertical angles as core network branches, and constructs a three-component coupled total loss to jointly optimize coordinate error and pinhole imaging consistency in an end-to-end manner under strict object-prior-free single-RGB input constraints, which constitutes the core methodological novelty of this work.

To avoid ambiguity between reused public modules and original contributions, we clarify the boundary of novelty here. The front-end pre-perception module consisting of YOLOv11 detector and DepthAnything-V2 depth predictor is mature off-the-shelf tooling for feature extraction, which is not regarded as our innovation. The three genuine novel components proposed in this paper cover the specially designed physical intermediate layer embedded in MLP-ResNet for closed-loop geometric feedback, the adaptive multi-dimensional physical constraint loss with piecewise angular penalty, and the cross-coupling loss term that mitigates the optimization trade-off between spatial error and angular deviation. Subsequent ablation experiments further quantify that these three proprietary modules jointly deliver significant localization accuracy gains that cannot be achieved by existing physics-guided monocular methods.

### 2.2. Summary of Existing Methods

The above literature analysis reveals three research gaps that limit the performance and generalization of existing single-view 3D localization methods. First, no existing paradigm is capable of object-prior-free single-view metric localization. All mainstream methods rely on mandatory auxiliary conditions to achieve metric (X,Y,Z) estimation, including predefined target dimensions, explicit 3D object models, category-based statistical priors, or multi-view triangulation principles. Specifically, PnP-based methods depend on precise object 3D models, SfM and SLAM pipelines require sequential camera motion for multi-view geometry, 3D bounding box detectors rely on object dimension priors, and early fusion methods are constrained by category-specific statistical information. Second, existing single-view localization solutions fail to achieve end-to-end physically consistent optimization without triangulation. Early fusion methods adopt a decoupled pipeline that cascades depth estimation and geometric projection, leading to unavoidable error accumulation and degradation in final localization accuracy. In contrast, pure deep learning regression methods completely discard geometric constraint supervision, resulting in severe coordinate drift and poor physical consistency under complex scene variations. None of the above methods embed explicit camera geometric rules into network learning objectives for unified single-view optimization. Third, prevailing single-view localization frameworks lack interpretable geometric constraint mechanisms. Current methods fail to construct explicit intermediate representations for geometric consistency enforcement; even the few approaches that introduce physical constraints treat them as implicit black-box regularization terms, rather than leveraging clear, differentiable geometric relationships derived from the single-view pinhole camera model.

The proposed PC-IMVL framework is specifically designed to address the aforementioned technical gaps in a unified manner. Different from conventional prior-dependent and decoupled optimization schemes, our method realizes direct single-view metric coordinate regression, which only requires camera intrinsic parameters and a single RGB image for accurate (X,Y,Z) estimation without relying on any object-specific priors or multi-view triangulation. Furthermore, PC-IMVL achieves thorough end-to-end physical constraint embedding by introducing differentiable geometric intermediate variables, including distance *d*, horizontal angle $\theta_{h}$, and vertical angle $\theta_{v}$, to enforce pinhole camera geometric consistency throughout the entire training process, thereby eliminating the error accumulation issue of decoupled pipelines and removing the demand for multi-view geometric verification. More importantly, the learned geometric intermediate variables carry explicit physical significance, constructing an interpretable constraint mechanism for single-shot localization predictions. This design not only enhances the physical rationality of network outputs but also facilitates model debugging and practical safety verification for real-world robotic localization applications.

## 3. Method

### 3.1. Model Architecture

**Figure 1:**
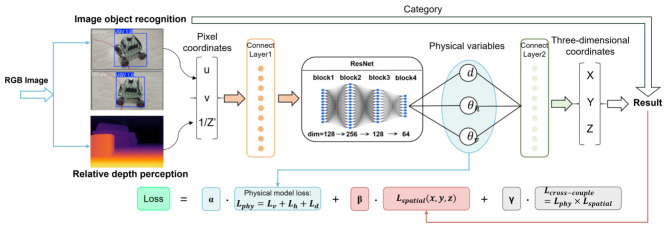
The model architecture of PC-IMVL.

Figure 1 presents PC-IMVL for indoor short-range scenes. Built on deep learning and physical constraints, it processes RGB images through four stages to output 3D coordinates (X,Y,Z). Key innovations include a physics-constrained loss function embedding physical logic end-to-end to avoid error accumulation and balance performance, and a closed-loop mechanism for real-time error correction ensuring physical consistency. The workflow involves RGB input, image recognition providing target category and pixel coordinates (*u*, *v*), relative depth estimation, feature fusion via MLP-ResNet-Physical Layer-MLP layers, and 3D coordinate generation through physics-constrained loss optimization.

### 3.2. Spatial Coordinate Prediction Network

PC-IMVL consists of a pre-perception module and a spatial coordinate prediction network, as shown in Figure 1. The pre-perception module includes image recognition [24] and relative depth prediction parts [26], completing target recognition and 3D reconstruction work; the spatial coordinate prediction network is the core of the PC-IMVL method, composed of MLP-ResNet-Physical Layer-MLP, realizing local feature extraction and global feature fusion. The network input is the target center pixel coordinates (u,v) output by the image recognition module and the relative depth $1/Z^{\prime }$ output by the relative depth perception module, and the output is the target spatial coordinates (X,Y,Z).

### 3.3. Physics-Constrained Loss Function

To mitigate error propagation and physical inconsistency in indoor short-range monocular object localization, we propose a multi-dimensional adaptive loss function with three core mechanisms: hierarchical spatial error penalization, physics-constrained loss optimization, and multi-objective cross-coupling. As a pivotal implementation framework for this study’s innovations, this loss function ensures high-precision 3D localization while embedding the pinhole camera imaging model as the physical constraint, achieving dual guarantees for localization accuracy and physical plausibility. The total loss function is formulated as:

(4) $$L_{\mathrm{total}}=\alpha L_{\mathrm{phy}}+\beta L_{\mathrm{spatial}}+\gamma L_{\mathrm{couple}},$$

where α, β, and γ denote the weighting coefficients for spatial loss, physics-constrained loss, and cross-coupling term, respectively. $L_{\mathrm{spatial}}$ represents the output positional error loss component, $L_{\mathrm{phy}}$ encapsulates the physical constraint loss derived from pinhole camera imaging principles, and $L_{\mathrm{couple}}$ is the cross-coupling term between output error and model-induced error, facilitating synergistic optimization of multiple objectives. The detailed formulation of each component is elaborated as follows:

#### 3.3.1. Physical Constraint Loss

**Figure 2:**
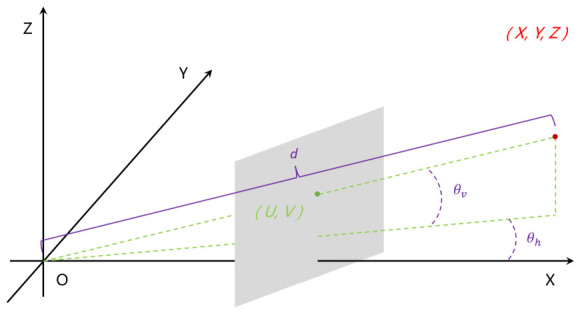
Schematic diagram of the physical model of a pinhole camera.

This paper designs a physically constrained loss based on the pinhole camera imaging model, as shown in Figure 2, assigning physical meanings to some nodes in the network by selecting intermediate variables such as distance *d*, horizontal angle $\theta_{h}$, and vertical angle $\theta_{v}$, and improves the numerical stability and physical rationality of the network through physical constraints. The mathematical expression is:

(5) $$L_{\mathrm{phy}}=L_{d}+L_{\theta_{v}}+L_{\theta_{h}},$$

where distance loss $L_{d}$ is defined as:

(6) $$L_{d}=d-d_{real}$$

Vertical angle loss $L_{\theta_{v}}$ and horizontal angle loss $L_{\theta_{h}}$ share the same form:

(7) $$L_{\theta }=\mathrm{ratio}\text{\_}\mathrm{err}\text{\_}f\left(\Delta \theta \right)\cdot smoothL1\left(\Delta \theta ,0.1\right),$$

where $\Delta \theta =|\theta_{\mathrm{pred}}-\theta_{\mathrm{gt}}|$ is the absolute angular difference, smoothL1(x,σ) is the smooth L1 loss that avoids gradient discontinuity at zero, and smoothing threshold ⊃ = 0.1:

(8) $$smoothL1\left(x,\sigma \right)=\left\{\begin{array}{cc} 0.5x^{2}/\sigma , & |x|<\sigma ,\\ |x|-0.5\sigma , & |x|\geq \sigma , \end{array}\right.$$

and ratio_err_f(x) is the angular difference segmented penalty function designed for precise optimization of small angles and rapid correction of large angles:

(9) $$\mathrm{ratio}\text{\_}\mathrm{err}\text{\_}f\left(x\right)=\left\{\begin{array}{cc} 33.333\cdot x^{2}, & x<0.03\;(\approx 3.43{}^{\circ}),\\ 2.0\cdot x-0.03, & x\geq 0.03. \end{array}\right.$$

When the angular difference x<3.43°, a quadratic penalty is used. The gradient approaches 0 as the error decreases, ensuring fine fitting of small angular deviations. When x≥3.43°, a linear penalty with a constant gradient of 2.0 is applied, achieving rapid correction of large angular deviations and balancing training efficiency with fitting accuracy.

#### 3.3.2. Spatial Loss

Addressing the problem where traditional single dominant errors (such as Euclidean distance) can easily mask single-axis deviations, this term combines 3D total error and single-axis component errors, with a bias-adaptive amplification function for fine small-error fitting and targeted large-error correction. Its mathematical expression is as follows:

(10) $$\begin{array}{cc} L_{\mathrm{spatial}}= & \;{\mathrm{MAB}}_{xyz}\cdot \mathrm{oursig}\left({\mathrm{MAB}}_{xyz}\right)\\ \; & +\sum_{k\in \{x,y,z\}}\left|\Delta k\right|\cdot \mathrm{oursig}\left(|\Delta k|\right), \end{array}$$

where ${\mathrm{MAB}}_{xyz}$ is the Euclidean distance error in the 3D space, Δk (k∈{x,y,z}) are absolute single-axis errors and oursig(x) is the bias-adaptive amplification function:

(11) $$\mathrm{oursig}\left(x\right)=\left\{\begin{array}{cc} 1.0, & x\leq 0.5,\\ \log(1+20\cdot (x-0.5)), & x>0.5. \end{array}\right.$$

For x≤0.5, the penalty weight is 1.0 to ensure stable gradients and avoid over-correction. For x>0.5, logarithmic growth avoids training oscillations while applying targeted constraints to samples with large devices.

#### 3.3.3. Cross-Coupling Loss

To alleviate the trade-off dilemma arising from independent optimization of output loss and physics-constrained loss, we introduce a cross-coupling mechanism that enforces joint optimization of both error modalities. This effectively eliminates physically implausible predictions where spatial proximity is achieved at the expense of violating physical imaging constraints. The mathematical formulation is given by:

(12) $$L_{\mathrm{couple}}=L_{\mathrm{phy}}\cdot L_{\mathrm{spatial}}$$

This coupling term imposes amplified penalties on samples exhibiting both significant spatial errors and substantial angular deviations, compelling the model to simultaneously satisfy the dual constraints of 3D localization accuracy and physical consistency during the optimization process. The proposed loss function achieves performance improvement through a triple mechanism:

1. Hierarchical error penalization: The spatial error component balances fine-grained fitting precision for small errors and efficient correction for large deviations.
2. Physics-informed constraint embedding: Addresses the issue of numerical optimization lacking physical interpretability by integrating imaging principles.
3. Multi-modal cross-coupling: Ensures concurrent optimization of localization accuracy and physical plausibility through synergistic error minimization.

Experimental verification shows that this loss function effectively suppresses error accumulation and improves model localization robustness in complex indoor scenes.

### 3.4. Training Strategy

The model is trained with the AdamW optimizer (weight decay $=1\times 10^{-4}$, batch size =16, total epochs =10,000, initial learning rate $=1\times 10^{-3}$) to prevent overfitting. We adopt an adaptive learning rate scheduling strategy with a decay patience of 5 epochs and a decay factor of 0.5. Specifically, when the loss shows no significant improvement for 5 consecutive epochs, the learning rate is reduced to 50% of its current value. The loss weight parameters are set as follows: spatial loss weight α=0.1, angular ratio loss weight β=0.2, and cross-coupling term weight γ=0.5. During training, we monitor loss stagnation in real-time, dynamically save the optimal model, and validate for fine-tuning to ensure generalization capability.

## 4. Experiments and Results

### 4.1. Experimental Design

**Figure 3:**
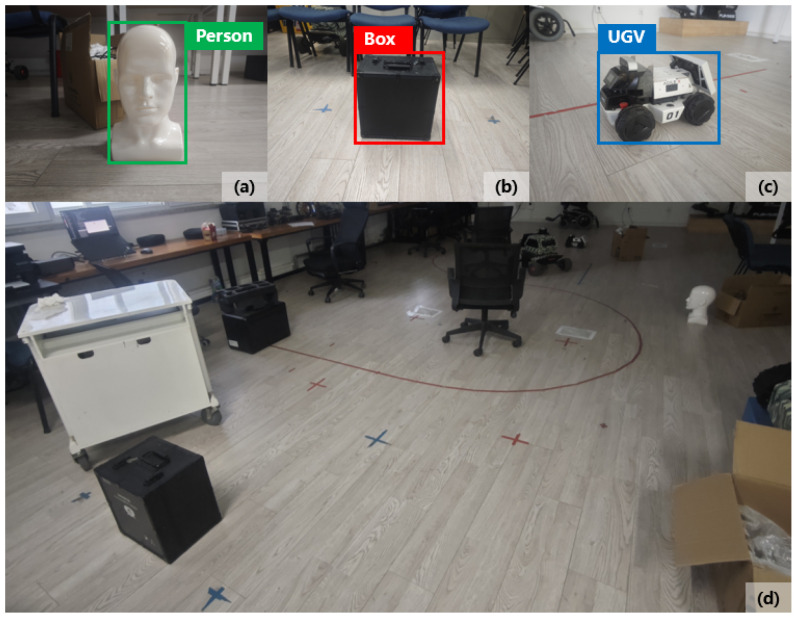
Illustration of visual objects, including (**a**) person, (**b**) box and (**c**) UGV, and (**d**) our experimental environment.

The dataset adopted in all experiments contains a total of 17,651 valid RGB image samples captured in a 12 m × 7 m × 3 m laboratory indoor environment, where ambient illumination is maintained within the range of 50 lux to 200 lux to reproduce typical dim and cluttered working scenes for miniature embedded indoor robots. The collected data covers continuous target-camera distances from 1 m to 6 m, with abundant cases of partial object occlusion and uneven lighting fluctuations that form the core challenging test conditions of our localization task. Three typical interactive targets for indoor robotic systems are fully annotated across all samples, namely person, unmanned ground vehicle (UGV) and storage box, covering deformable human bodies and rigid objects of different geometric sizes. Figure 3 illustrates example targets: (a) Person, (b) Box, and (c) UGV. The experimental scenes are shown in Figure 3d.

Each data sample stores aligned RGB frames, target center pixel coordinates, pixel-level relative depth maps and high-precision metric 3D coordinates under the camera coordinate frame, and all 3D ground-truth labels are calibrated using an optical tracking system to achieve centimeter-level annotation accuracy. Standard data augmentation strategies including random brightness jitter and small-angle rotation are applied during model training to improve the network’s robustness against complex indoor lighting variations, and the entire dataset is split into training, validation and test subsets at a ratio of 7:1.5:1.5 to guarantee balanced sample distribution for stable ablation and comparative experiments. Widely used public indoor datasets such as SUN RGB-D, ScanNet and ARKitScenes are not suitable for the task proposed in this paper due to fundamental mismatches in data collection equipment, annotation types and scene distribution. These benchmarks are primarily developed for dense scene reconstruction and general indoor semantic segmentation, and most data are captured by RGB-D sensors or iPad LiDAR devices instead of pure monocular RGB cameras, creating an obvious domain gap with our single-view monocular input pipeline. Their annotated objects are dominated by static large furniture while lacking sufficient labeled samples of dynamic targets such as walking humans and mobile UGVs, which are the key perception objects for indoor service robots. Moreover, these public datasets cannot provide complete instance-level metric 3D coordinate labels matched with single RGB frames, which are essential for training the spatial coordinate regression branch and multi-component physical loss function of PC-IMVL. Most samples within public indoor datasets focus on long-distance indoor scenes rather than the 1–6 m short-range working range of our robotic localization task, while outdoor benchmarks KITTI and nuScenes target autonomous driving scenarios with completely different object scales and camera distance distributions, making them unable to support fair evaluation of our indoor short-range localization method. For the above reasons, we construct a task-specific custom dataset that fully matches the single-view, prior-free, close-range indoor localization setting to ensure consistent experimental conditions and uniform high-quality annotations for reliable model training and comparative analysis.

All model training experiments are implemented on a high-performance computing platform equipped with an Intel Core i9-13900K CPU, 64 GB of system RAM, and an NVIDIA RTX 4060 GPU with 8 GB VRAM. The software environment for training is built based on CUDA 11.8, PyTorch 2.0, and Python 3.10 to ensure stable model training and efficient GPU acceleration. For practical online deployment, we adopt the NVIDIA Jetson AGX Orin embedded platform with 64 GB RAM and no independent dedicated GPU, where the runtime environment is configured with CUDA 11.4 and Python 3.8, matching the lightweight and low-power requirements of real-world robotic embedded scenarios. The online deployment experiments are fully executed on this Jetson AGX Orin edge device, and the embedded runtime built with CUDA 11.4 and Python 3.8 meets the low-latency inference demands of lightweight indoor robots.

#### 4.1.1. Experiments

We design three sets of comparative experiments to evaluate the performance of PC-IMVL against the Early Fusion Method (EFM) and pure Deep Learning-driven method (DL). All methods use identical pre-perception (YOLOv11+DepthAnythingV2) and single-view RGB input (1280×1024). Average Execution Time (AET) is measured as end-to-end latency from image input to 3D coordinate output for one inference round.

We provide detailed implementation configurations for the proposed PC-IMVL method and comparative baseline models for rigorous and fair evaluation. The full PC-IMVL model incorporates the complete physics-constrained total loss $L_{\mathrm{total}}$ defined in Equation (4), and explicitly predicts physically interpretable intermediate variables $\left(d,\theta_{h},\theta_{v}\right)$ embedded with inherent pinhole camera geometric priors. For the early fusion (EFM) baseline, we adopt a powerful pipeline combining DepthAnything-V2 [10] for accurate monocular depth estimation and YOLOv11 [24] for reliable 2D object detection, where standard pinhole projection together with category-averaged dimension priors (1.7 m for persons, 0.5 m for boxes, and 0.3 m for UGVs) is utilized to alleviate scale ambiguity. This configuration constructs a state-of-the-art EFM variant that excludes instance-level prior information. To further validate the effectiveness of the embedded physical constraints, we develop a pure deep learning (DL) baseline ablated from our PC-IMVL framework. This baseline removes the physical constraint loss $L_{\mathrm{phy}}$ and all geometric intermediate variables and only employs the spatial loss $L_{\mathrm{spatial}}$ to implement end-to-end direct (X,Y,Z) coordinate regression. Notably, the ResNet-34 backbone and MLP regression heads are strictly consistent with the full PC-IMVL model to eliminate structural differences and ensure a fair ablation comparison.

The experimental platform is an indoor unmanned vehicle equipped with a monocular camera (resolution 1280×1024). Experiments were conducted in a 12m×7m×3m indoor scene (50–200 lux) with target-camera distances ≤ 6 m and vehicle speeds ≤ 0.4 m/s. Offline, simulated, and ablation experiments utilized the training hardware, while real-world experiments were deployed on an edge computing platform (NVIDIA Jetson AGX Orin), employing YOLOv11+DepthAnythingV2 as the pre-perception module for all three methods to ensure fair comparison.

#### Offline Experiment

Targets were detected and their coordinates predicted across four distance intervals: 1–2 m, 2–3 m, 3–4 m, and 4–5 m. Absolute error (linear distance between predicted and ground-truth coordinates, AE) and relative error (absolute error divided by true distance, RE) were used to evaluate PC-IMVL’s mitigation of error accumulation.

#### Simulated Experiment

In the experimental environment, the robot operated to provide real-time video streams and odometry data, with positions recorded in map coordinates. AE and horizontal Viewing-Angle Error (VAE) were used to evaluate models.

#### Ablation Experiment

The dataset is partitioned into training, validation, and test subsets at a 7:1.5:1.5 ratio. Ablation experiments on the loss function systematically evaluate the contribution of each component to model localization performance.

#### Real-World Experiment

Deployed on an edge computing platform (NVIDIA Jetson AGX Orin, Ubuntu 20.04) with ROS, the system performed target detection and mapping in the aforementioned indoor environment with target positions at UGV (−1.5,0,0), person (2,−2,0), and box (5,−1,0) meters. Performance was evaluated using AE, Sample Variance (SV), and Average Execution Time (AET) based on the mapping results.

### 4.2. Offline Experiment Results

**Figure 4:**
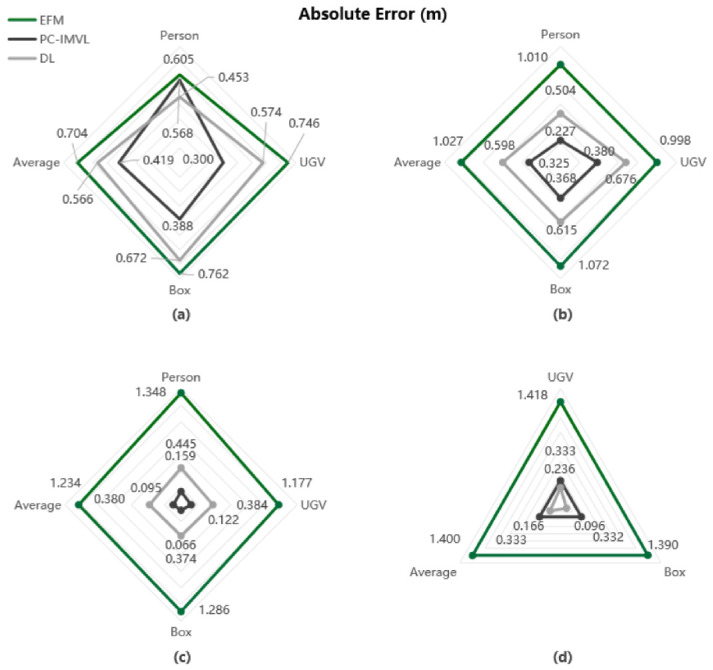
Offline experimental results of the absolute errors across different targets at different distances: (**a**) 1–2 m, (**b**) 2–3 m, (**c**) 3–4 m, and (**d**) 4–5 m.

As shown in Figure 4, our experiment demonstrates that PC-IMVL outperforms EFM and DL across all distances and target categories with superior distance adaptability. At 1–2 m, PC-IMVL achieves minimal AE: 0.568 m (Person), 0.330 m (UGV), and 0.360 m (Box), significantly outperforming EFM’s 0.859 m for Person. At 2–3 m, PC-IMVL retains low AE (0.195 m for Person, 0.200 m for UGV) while EFM’s Person error escalates to 1.011 m. At 3–4 m, PC-IMVL achieves its minimum AE of 0.120 m for UGV, whereas EFM/DL lose practical precision (e.g., EFM’s 1.348 m for Person). At 4–5 m, PC-IMVL maintains low error (0.165 m for Person, 0.330 m for Box) while EFM/DL peak (EFM’s 1.418 m for UGV). PC-IMVL exhibits consistent AE superiority with robust long-distance stability, whereas EFM/DL show distance-dependent error escalation, particularly for Person targets.

**Figure 5:**
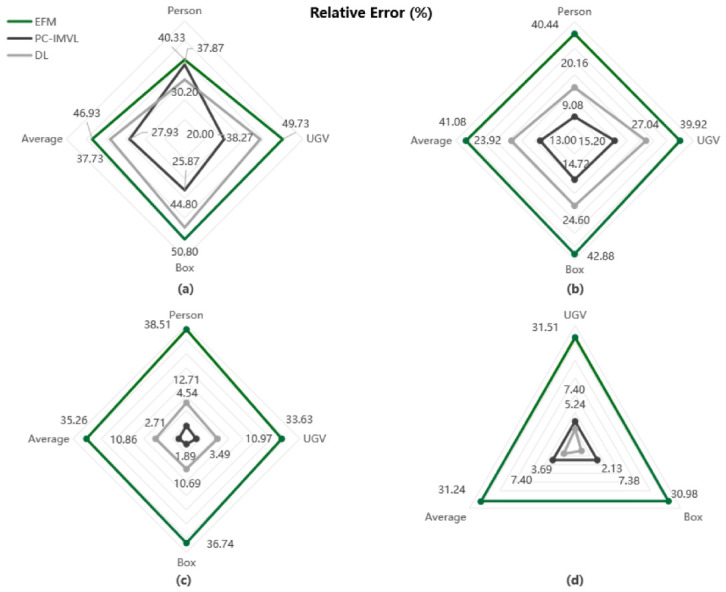
Offline experimental results of the relative errors across different targets at different distances: (**a**) 1–2 m, (**b**) 2–3 m, (**c**) 3–4 m, and (**d**) 4–5 m.

In addition, as shown in Figure 5, across 1–5 m, PC-IMVL achieves consistently lower relative error (RE) for all targets (14.05–29.11%) with strong long-distance stability. In contrast, EFM/DL exhibit distance-dependent RE escalation (e.g., 48.15–48.34% for Person, 37.13% for UGV), demonstrating poor precision and high sensitivity to Person targets. These results demonstrate PC-IMVL’s comprehensive advantages in accuracy and generalization, effectively addressing error accumulation.

### 4.3. Simulated Experiment Results

**Figure 6:**
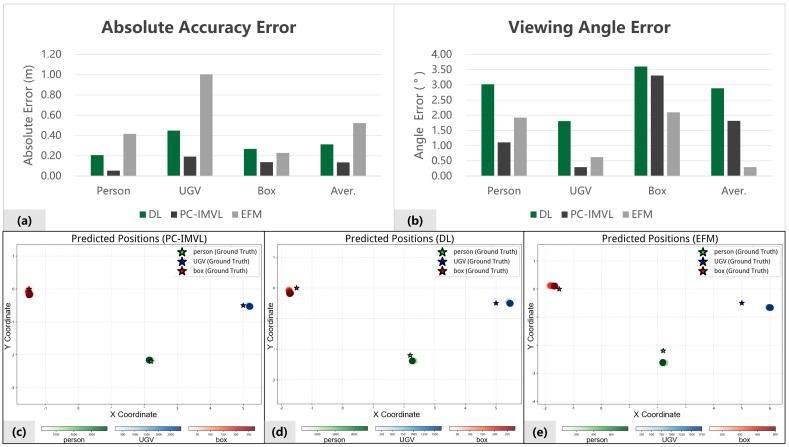
Simulated experiment results. (**a**) Absolute error, (**b**) viewing angle error, (**c**–**e**) the prediction scatter distributions of different methods.

The superiority of PC-IMVL for indoor dynamic localization is quantitatively validated in terms of absolute error (AE) and viewing angle error (VAE). As illustrated in Figure 6a, PC-IMVL achieves the lowest AE across all tested object categories, including persons, UGVs, and boxes. In particular, our method yields a substantial accuracy improvement over the EFM baseline and delivers superior performance compared to the pure DL baseline for human targets. For UGV localization, PC-IMVL maintains an average error of approximately 0.2 m, while both EFM and DL methods suffer from significantly larger errors exceeding 1.0 m. Overall, PC-IMVL reduces the average absolute error to around 0.2 m, corresponding to a 50% accuracy improvement over the DL baseline (approximately 0.4 m) and an 80% reduction compared with the EFM baseline (approximately 1.0 m). These results verify that the embedded physical constraints effectively correct spatial localization deviations for common textured indoor objects. In terms of horizontal viewing angle error, Figure 6b shows that PC-IMVL maintains a stable average error of ∼1°, which favorably surpasses the DL baseline (∼2.5°) and the EFM baseline (∼3.0°), with an especially remarkable performance margin of only ∼0.5° for UGV and box targets. The prediction scatter distributions visualized in Figure 6c–e further support these conclusions. The predictions generated by PC-IMVL are tightly clustered around the ground-truth positions, whereas DL and EFM predictions present severe spatial deviations and inconsistent geometric outputs. Collectively, these consistent improvements in both positional and angular accuracy demonstrate that embedding explicit physical constraints during training can effectively compensate for the inherent physical irrationality and accuracy limitations of conventional DL regression and decoupled EFM pipelines, thereby verifying the technical effectiveness of the proposed PC-IMVL for dynamic indoor single-view metric localization.

### 4.4. Ablation Experiment Results

The results in Table 2 illustrate the distinct and complementary roles of individual loss components and their combined effects on localization performance. The standalone spatial loss $L_{\mathrm{spatial}}$ achieves a competitive absolute error of 0.265 m, establishing a reliable foundation for positional regression. In contrast, the physical constraint loss $L_{\mathrm{phy}}$ yields a large positional error of 21.992 m when applied independently but produces a favorable angular error of 5.71°, demonstrating its unique superiority in optimizing orientation estimation. The combination of $L_{\mathrm{spatial}}$ and $L_{\mathrm{couple}}$ further reduces the absolute error to 0.252 m yet increases the angular error to 9.00°, revealing an inherent trade-off between positional and angular optimization. Benefiting from the complementary properties of all three components, the complete loss formulation integrating $L_{\mathrm{spatial}}$, $L_{\mathrm{phy}}$, and $L_{\mathrm{couple}}$ achieves the lowest angular error of 4.71° while maintaining a near-optimal absolute error of 0.261 m. These ablation results confirm that the collaborative integration of multiple loss terms effectively alleviates the positional-angular trade-off encountered by individual or partial combinations. Such multi-module complementary optimization is therefore essential for comprehensively balancing positional and angular accuracy, which underpins the superior overall performance of the proposed PC-IMVL framework.

### 4.5. Real-World Experiment Results

**Figure 7:**
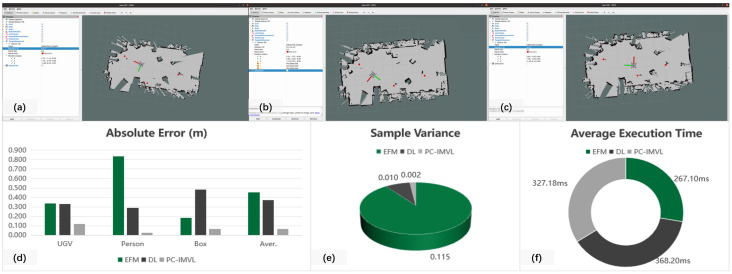
Real-world experimental results across different methods (**a**) EFM, (**b**) DL, and (**c**) PC-IMVL. Performance metrics include (**d**) absolute error, (**e**) sample variance, and (**f**) average execution time.

As visualized in Figure 7, real-world experimental results demonstrate that the proposed PC-IMVL method achieves substantial performance gains over both the EFM and DL baselines in terms of absolute error (AE) and spatial variance (SV), delivering superior localization precision and environmental robustness. In terms of average execution time (AET), PC-IMVL presents a well-balanced computational efficiency, and it exhibits a clear efficiency improvement compared with the computationally cumbersome DL baseline. These consistent real-world observations further validate the overall superiority of PC-IMVL in achieving accurate, robust, and efficient single-view metric localization in practical scenarios.

### 4.6. Discussion

This paper addresses the critical issues of error accumulation and physical inconsistency inherent to indoor short-range monocular visual object localization via the proposed PC-IMVL framework. By embedding physical geometric constraints into the multi-component loss function and optimizing the spatial coordinate prediction backbone, PC-IMVL delivers physically consistent, high-precision 3D localization without relying on object priors or extra sensing hardware. Consistent offline, simulation and real-world embedded experiments validate that PC-IMVL outperforms mainstream monocular localization baselines in terms of absolute localization error, sample prediction variance and horizontal viewing angle error, achieving superior localization accuracy, environmental robustness and deployment compatibility for edge robotic platforms.

It is worth clarifying that the “object-prior-free” property of PC-IMVL only excludes explicit target scale templates and 3D object models for inference and does not exclude general hardware calibration parameters, pre-trained feature extraction modules or supervised training labels, which are universal basic components of standard monocular vision pipelines. Despite the promising performance achieved within indoor short-range scenarios, the current implementation still carries application limitations, particularly regarding generalization to open outdoor environments. The core pinhole camera geometric model and physics-guided loss function of PC-IMVL are universal imaging rules without inherent indoor-only restrictions, which theoretically enables outdoor object localization. Even so, several practical obstacles should be addressed before stable outdoor deployment. Severe dynamic outdoor lighting, including intense sunlight, backlight and irregular shadows, degrades the reliability of monocular relative depth estimation, weakening the regularization effect of our physical consistency loss. In addition, outdoor targets generally stand far beyond the optimized 1–6 m working range of this work; subtle angular variations at long distances reduce the sensitivity of angular loss to spatial localization deviations. Unstructured outdoor clutter such as vegetation and complex buildings also introduces irrelevant noise features that interfere with target geometric feature extraction and degrade overall localization stability. To resolve these barriers, we will carry out targeted follow-up research dedicated to outdoor-adaptive localization: we plan to build mixed indoor–outdoor datasets covering diverse illumination and distance distributions, integrate multi-style illumination augmentation during model training, design a distance-adaptive weight adjustment strategy for physical loss terms, and refine the feature extraction branch to suppress background interference.

In the current experimental setup, all validation data are collected within a unified laboratory indoor space and cover three representative object classes (person, UGV, box) typical for indoor robotic interaction. While the dataset incorporates varied target distances, partial occlusion and moderate illumination fluctuations from 50 lux to 200 lux, further multi-scene and multi-category tests will help fully explore the generalization boundary of PC-IMVL across richer real-world indoor conditions. Real indoor spaces feature distinct room layouts, furniture arrangements and various unseen everyday objects that are not fully covered in the existing dataset, which motivates us to expand data coverage in follow-up work. We will collect samples from multiple types of indoor spaces such as offices, storage rooms and narrow corridors to diversify scene layouts, extend illumination coverage to cover ultra-dim and bright indoor environments, and introduce numerous unseen object categories including chairs, tables and daily appliances for cross-category generalization tests. Cross-dataset evaluation based on public multi-scene indoor benchmarks will also be conducted to further enrich the experimental evidence supporting the generalization capacity of our physics-guided localization pipeline.

Beyond outdoor generalization, multiple promising technical directions can be explored in subsequent research to further elevate the overall performance and engineering practicability of PC-IMVL. First, we will construct comprehensive simulation environments covering diverse extreme indoor scenes and combine reinforcement learning strategies to reduce dataset dependency and strengthen model robustness against unseen cluttered environments. Second, reinforcement learning-driven adaptive loss weighting mechanisms will be explored to realize dynamic, data-aware parameter tuning throughout the training process. Third, knowledge distillation will be utilized to design lightweight integrated architectures that merge pre-perception modules and coordinate prediction branches, lowering computational overhead and facilitating broader lightweight embedded deployment. Furthermore, the proposed monocular framework can be extended to binocular camera systems to further boost ranging precision, where stereo disparity can serve as an extra geometric prior to fundamentally eliminate monocular scale ambiguity, at the cost of sacrificing the low-cost, compact deployment advantages of single-camera solutions. To further validate the cross-dataset generalization performance of PC-IMVL, multiple public indoor datasets would be introduced in subsequent research to conduct comprehensive cross-domain evaluation.

## 5. Conclusions

This work presents PC-IMVL, a physics-constrained monocular visual localization framework that addresses the key limitations of existing single-view metric localization methods, including prior dependence, geometric inconsistency, and low interpretability. Unlike conventional multi-view triangulation, depth-based early fusion, and purely data-driven regression schemes, PC-IMVL embeds explicit pinhole geometric constraints into end-to-end network optimization. By leveraging interpretable physical intermediate representations and a collaborative multi-component loss function, our method enables object-prior-free single-shot metric coordinate estimation without requiring object category statistics, 3D templates, or multi-view parallax, thus ensuring physically consistent localization predictions. Sufficient ablation studies validate the complementary roles of spatial, physical, and coupling losses, which effectively mitigate the trade-off between positional and angular optimization. Comparative tests and real-world dynamic experiments further verify that PC-IMVL achieves superior precision, angular stability, and robustness against baseline methods while maintaining feasible computational efficiency for embedded deployment.

## Figures and Tables

**Table 1:** Comparisons of monocular 3D localization methods.

Method Type	3D Models Required	Dimensions Required	Multi-View Triangulation	Metric (X,Y,Z) Direct Output	Physical Constraints
PnP-based [4,17]	Yes	Yes	No	Yes	Post-hoc
SfM/SLAM [5,12]	No	No	Yes	Yes	Post-hoc
Stereo Methods [18]	No	No	Yes	Yes	Explicit
3D Bounding Box [6,7]	No	Yes ^†^	No	No ^‡^	Implicit
Early Fusion [15,19]	No	Yes (category)	No	Yes	Post-hoc
Pure DL Regression [20]	No	No	No	Yes	None
PC-IMVL (Ours)	No	No	No	Yes	End-to-end

^†^ Required for metric scale recovery. ^‡^ Outputs pose + dimensions; coordinates need post-processing.

**Table 2:** Ablation experiment results.

Loss Configuration	AE (m)	VAE (°)
$L_{spatial}$	0.265	7.57
$L_{phy}$	21.992	5.71
$L_{couple}$	0.373	4.85
$L_{spatial}+L_{phy}$	0.299	5.24
$L_{spatial}+L_{couple}$	0.252	9.00
$L_{phy}+L_{couple}$	0.617	5.44
$L_{spatial}+L_{phy}+L_{couple}$	**0.261**	**4.71**

## Data Availability

The original contributions presented in this study are included in the article. Further inquiries can be directed to the corresponding author.
